# Vaccinations in Paediatric Solid Organ Transplant Candidates and Recipients

**DOI:** 10.3390/vaccines12090952

**Published:** 2024-08-23

**Authors:** Valeria Casotti, Paola Stroppa, Michela Bravi, Alessandra Tebaldi, Alessandro Loglio, Mauro Viganò, Stefano Fagiuoli, Lorenzo D’Antiga

**Affiliations:** 1 Paediatric Hepatology, Gastroenterology and Transplantation, Child Health Department, ASST Papa Giovanni XXIII, 24127 Bergamo, Italy; pstroppa@asst-pg23.it (P.S.); mbravi@asst-pg23.it (M.B.); ldantiga@asst-pg23.it (L.D.); 2 Infectious Diseases Unit, ASST Papa Giovanni XXII, 24127 Bergamo, Italy; atebaldi@asst-pg23.it; 3 Division of Gastroenterology, Hepatology and Transplantation, ASST Papa Giovanni XXIII, 24127 Bergamo, Italy; aloglio@asst-pg23.it (A.L.); mvigano@asst-pg23.it (M.V.); sfagiuoli@asst-pg23.it (S.F.); 4 Department of Medicine and Surgery, University of Milano-Bicocca, 20126 Milano, Italy

**Keywords:** vaccinations, solid organ transplant (SOT), paediatric, infections

## Abstract

Solid organ transplant (SOT) candidates and recipients are a fragile population, in which the presence of a pre-transplant disease leading to organ insufficiency and the post-transplant immunosuppressive treatment expose them to an increased risk of infectious diseases. The best intervention to guarantee efficient prevention of infections, with optimal cost–benefit ratio, is represented by vaccination programs; however, the response to vaccines needs that the immune system maintains a good function. This is even more relevant at paediatric age, when specific immunological conditions make transplant candidates and recipients particularly vulnerable. Paediatric patients may be naïve to most infections and may have incomplete immunization status at the time of transplant listing due to their age. Moreover, the unaccomplished development of a mature immune system and the immunosuppressive regimen adopted after transplant might affect the efficacy of post-transplant vaccinations. Therefore, every effort should be made to obtain the widest vaccination coverage before the transplantation, whenever possible. This review reports the most relevant literature, providing information on the current approach to the vaccinations in paediatric SOT candidates and recipients.

## 1. Introduction—General Indications

Despite the progress in transplant medicine management, infectious diseases still represent a major cause of morbidity and mortality in solid organ transplant (SOT) recipients, in comparison with immunocompetent individuals.

Vaccination is the best-known intervention to prevent infectious diseases, in terms of both effectiveness and cost–benefit; however, the response to vaccines is strictly related to a functioning immune system [1,2].

This is particularly true for paediatric transplant recipients, regardless of the organ graft type. Indeed, these patients have a higher incidence of vaccine-preventable infections (VPIs) compared to the general population and to adult recipients, and this is mainly due to the following risk factors:

- Lack of previous immunity from natural infection exposure;
- Incomplete immunization schedule at the time of transplantation;
- Incomplete or inadequate response to vaccines due to the post-transplant immunosuppressive treatment;
- Progressive waning of the protective antibody titre, occurring after transplantation [3].

As an example, we report the results of a multicentre cohort study involving 6980 paediatric SOT recipients at a Paediatric Health Information System centre in the USA. In this observational study, 16% of patients were hospitalized at least once in the first five years after transplant because of VPIs, and the overall mortality for these conditions was 1.7%. The most common VPIs observed were influenza-virus (40% of cases), rotavirus (19%), chicken-pox (11%), *Streptococcus pneumoniae* (10%) and respiratory syncytial virus (10%). 

The patients at major risk for hospitalization with VPIs were the following:

- Children who received transplants when younger than two years;
- Transplant recipients of lung, intestine, heart and multivisceral organs.

This is probably because, usually, children undergoing transplant before two years of age are less likely to have completed their vaccination schedule at the time of transplantation, whereas children receiving lung, heart, intestine and multivisceral grafts are treated with the highest levels of immunosuppression to prevent rejection [4,5].

Several case reports described severe and devastating illnesses, as well as graft failures and rejection, in unvaccinated children after renal and liver transplant, secondary to measles, mumps or primary varicella-zoster infections [6].

So far, although current recommendations emphasize the importance of prompt vaccinations in SOT candidates before the transplantation, vaccine coverage studies have demonstrated that this aim is far from being realized [1].

The incomplete immunization status of children affected by chronic diseases that are candidates for a SOT has several reasons:

- The complex primary medical problems, with frequent hospitalizations and insufficient opportunities, for which a child is considered well enough to proceed with immunizations;
- The fact that primary care practitioners sometimes consider the patient’s underlying chronic condition as a contraindication for immunization;
- The sub-optimal antibody responses in chronic liver and renal diseases;
- The parental hesitancy or aversion against vaccinations [2,7,8].

Transplant candidates affected by end-stage organ disease, and transplant recipients in treatment with immunosuppressive drugs, may not be able to mount adequate immune responses to vaccines, as compared to healthy individuals; moreover, they may show a decrease in antibody titres, more rapid than immunocompetent subjects [2,8].

Several studies have shown the under-immunization status of paediatric SOT, both before and after transplantation.

In a study from the US, less than 30% of liver transplant recipients were up-to-date on age-appropriate immunizations at the time of transplant [8].

A recent European study, including 430 children from five liver transplant centres, revealed a global under-immunization status: 80% were vaccinated against tetanus-diphtheria-pertussis (DTaP), poliomyelitis (PV), and haemophilus influenzae type B (HiB), compared to national standards; 84% were age-appropriately vaccinated for hepatitis B virus (HBV), 16.6% for rotavirus, 60% for pneumococcal infection, 81% for measles-mumps and rubella (MMR) and 65% for varicella-zoster virus (VZV) [9]. 

In another study, evaluating the immunization status of 90 paediatric liver transplant candidates, the percentage of patients with protective antibody titres was the following: 80% for poliomyelitis, 65.6% for rubella, 62.3% for diphtheria, 60% for tetanus, 57.7% for pertussis, 55.5% for measles, 42.2% for HBV and 36.7% for mumps [10].

Moreover, in a small study about 34 patients receiving an intestinal transplant, only DTaP, polio and HBV were administered correctly before transplant, but with low sero-conversion levels as compared to the healthy population [11].

Lastly, a study on 30 paediatric SOT recipients (kidney, liver, heart) showed that, at the time of SOT, vaccination status was completed only in a percentage of patients; in particular:

- 47% of patients had all recommended doses for their age of the 6-fold vaccine (containing DTaP, HBV, poliomyelitis and HiB), plus their *Streptococcus pneumoniae* conjugate vaccine and the MMR vaccine.
- 30% of children had partially complete vaccination status (lack of the 2nd dose of MMR);
- 23% had incomplete vaccination status [8,12].

In the pre-transplant period, several practical problems, difficult to overcome, can affect timely vaccination; on the other hand, concerns by both patients’ parents and medical staff about possible vaccine-induced side effects can cause hesitancy towards vaccinations in the post-transplantation period [1]. 

In addition, an important issue is the rising rate of vaccine hesitancy amongst the general population, leading to decreased herd immunity, which may no longer protect non-immune individuals, such as unvaccinated transplant recipients [13,14].

Considering all these evidences, every effort should be made to immunize patients prior to the transplantation, whenever possible and provided they are medically stable, and this is true for all the transplant programs available for paediatric age [7,15]. 

Both Infectious and Transplant Societies recommend that SOT candidates receive all age-appropriate vaccines based on the schedule for immunocompetent persons; this should occur ideally as early as possible over the course of their end-stage organ disease [5,16].

The vaccination status can be documented at the pre-transplant clinic visit, based on immunization records and screening serology. 

The general vaccination recommendations for children can be provided by different sources:

- World Health Organization (WHO): https://www.who.int/teams/immunization-vaccines-and-biologicals/policies/who-recommendations-for-routine-immunization---summary-tables (accessed on 20 July 2024).
- Centre for Disease Control and Prevention (CDC): https://www.cdc.gov/vaccines/ (accessed on 20 July 2024).
- The European Centre for Disease Prevention and Control (ECDC), providing country specific vaccination recommendations: https://vaccine-schedule.ecdc.europa.eu/ (accessed on 20 July 2024)

The suggested vaccination schedules from WHO and CDC, which can be applied to SOT candidates and recipients, are summarised in Table 1.

To obtain the best immunization status before the transplantation, all the inactivated vaccines should be administered at least 2 weeks before SOT, while all live vaccines should be given at least 4 weeks before SOT, to be sure that viral replication has ended prior to initiating immunosuppressive therapy [3].

Children with end-stage organ disease requiring transplantation who have not completed the vaccine schedule at the time of listing can receive vaccinations on an accelerated schedule [5,17,18]. If the vaccination schedule cannot be completed before the transplantation, continuation can occur in the post-SOT period. 

There is no clear consensus on how the vaccination schedule should restart; for sure, during the first 2 months after transplantation, when immunosuppression is at a higher level, vaccinations should be withheld, since there is a high likelihood of inadequate response. Most centres resume vaccinations approximately 3–6 months after transplantation: at that time, lower or minimum immunosuppression levels are attained [19,20,21]. 

After SOT, it is frequent to observe a significant depletion in protective antibody titres due to the immunosuppression; for this reason, the use of serologic tests can guide management: the evidence of waning immunity defines the need for booster doses of specific vaccines, necessary to guarantee optimal protection [3,6,15,21]. 

Previous studies, investigating inactivated and live attenuated vaccines in both adult and paediatric SOT recipients (renal, liver, heart, lung or mixed cohorts), reported neither severe adverse events nor transplant related complications (such as organ rejection) after vaccination [22,23]. It is now established that vaccines do not cause a higher risk of rejection; conversely, they are associated with a reduced risk of allograft loss or death after SOT [1,3]. 

At present, the option to administer live vaccines in the post-transplant setting is a matter of debate. So far, standard recommendations have stated that these vaccines should be avoided after transplant, for two main reasons:

- The possibility that the viral-strain pathogen can cause a severe or life-threatening infection in an immunocompromised patient;
- The possibility that patients cannot mount an adequate and protective immune response, due to the immunosuppressive treatment.

Despite this background, there are some studies and strong anecdotal evidence suggesting that in specific conditions, when certain transplant recipients are treated with minimal immunosuppression, live vaccines can be administered, and they have a good profile of safety and efficacy. 

In a consensus conference held in 2018, a series of recommendations were obtained, mostly based on data from paediatric kidney and liver transplants, vaccinated with MMR vaccine and monovalent varicella vaccine [13,24].

On the contrary, there are some live vaccines remaining contraindicated in all SOT recipients, including: oral Polio Vaccine (OPV), oral Salmonella typhi vaccine, smallpox (vaccinia), Bacillus Calmette–Guerin (BCG) and inhaled live attenuated influenza vaccine [7]. 

A special consideration shall be given for the more recent COVID-19 vaccines, developed to face the SARS-CoV-2 pandemic challenge, since the recommendations for different population categories may still change over time, according to evolving knowledge and the transfer of information between adulthood and paediatric age.

### 1.1. Vaccination in Healthcare Workers and Close Contacts

Healthcare personnel and close contacts, such as parents and family members, of paediatric transplant recipients, should be considered for the widest possible immunization. This approach can increase the vaccination coverage, providing a theoretical household herd immunity against VPIs for susceptible SOT patients. 

Household contacts of SOT recipients can safely receive the following:

- All the inactivated vaccines;
- Vaccination against influenza yearly, preferably with inactivated vaccine (IIV).
- Most of the live vaccines: MMR, varicella, Rotavirus (if infants are aged 2–7 months).

Some vaccine remains contraindicated, due to the risk of transmission to the SOT recipient of the vaccine-related disease: OPV, smallpox and live inhaled influenza vaccine [7,8,18,25]. 

### 1.2. Travel Vaccinations

SOT transplant recipients, both adults and paediatrics, are at higher risk of travel-related illnesses, as compared to the healthy population, from both communicable and non-communicable diseases. This is dependent on the intensity of immunosuppression, the travel destination and the likely sub-optimal response to pre-travel vaccines.

Up to 36% of SOT recipients are active in international trips. For these reasons, it is essential, for adult and paediatric transplant physicians, to know the potential travel-related infections their patients can acquire by travelling, and to suggest the necessary pre-travel vaccines, for both disease prevention and/or decrease in disease severity, in case of infection. Most of the indications overlap with those of the healthy population. The main diseases for which a vaccination has to be considered, sometimes as a suggested practice, sometimes as an obligation in case of travelling in high-risk areas, are the following: yellow fever, typhoid vaccine, Japanese encephalitis, rabies, cholera and BCG [16,26]. 

## 2. Methods

A PubMed search was performed with the following keywords: vaccines/vaccinations AND solid organ transplant/transplantation AND infections. We considered the most relevant papers referring to the SOT general population, then we focused on the SOT paediatric population, filtering the search by age (children 0–18 years).

## 3. Inactivated-Recombinant Vaccinations: Pre- and Post-Transplant Indications

All inactivated vaccines are considered safe in the paediatric population, both before and after solid organ transplantation. When no data are available on SOT candidates and recipients, the correct approach is to follow the recommendations made for the general population, according to the different national vaccination schedules (see Table 1) [25]. 

### 3.1. Tetanus and Diphtheria

Diphtheria and tetanus, combined with the pertussis vaccine (DTaP), is recommended to be administered before SOT, since it is safe and immunogenic in patients with end-stage liver and renal disease [21]. During pre-transplant evaluation, it is useful to check tetanus serology, regardless of the patient’s vaccination history, since it represents the best correlate to evaluate vaccine-induced protection after DTaP immunization [26]. 

After SOT, patients can continue and update immunization for tetanus and diphtheria by using the same schedule as healthy children. 

The measured long-term response for tetanus tends to persist in SOT recipients on standard immunosuppression, with a response rate ranging from 85–100%, similar to that observed in the healthy population. 

For diphtheria, short-term response rates ranged from 88.5% to 95%, with comparable response rates in SOT recipients and controls, whereas only 38–57% had adequate antibody titres one year after immunization [7]. 

After SOT, paediatric patients should be periodically monitored for post-immunization titres, with a suggested timing of every five years for tetanus and every two years for diphtheria. According to antibody assessment, they can receive booster doses of tetanus and diphtheria vaccines at regular intervals, which are demonstrated as safe and effective for SOT patients [7]. 

### 3.2. Pertussis

It is known that, in healthy and immunocompetent children, the acellular pertussis vaccine is considered safe and effective, but there are no data on SOT patients. For this reason, the best approach should be to make every effort to administer the pertussis vaccine before transplantation, according to the standard schedules. If pre-transplant immunization is incomplete or not feasible, it is reasonable to start or to resume the vaccination schedule between six months and one year after SOT [7]. 

### 3.3. Poliomyelitis

Immunization for poliomyelitis is recommended for all children, regardless of immune status, and Inactivated Polio Vaccine (IPV) can be part of their combined vaccinations.

SOT candidates and recipients should receive IPV, and they can follow exactly the standard schedule approved for the general population, either before or after transplantation. In the case of the administration of booster doses, SOT patients show similar antibody responses and vaccine efficacy as compared to healthy children [7].

### 3.4. Haemophilus Influenzae Type B (HiB)

SOT candidates and recipients should be vaccinated against HiB with a conjugate vaccine, by following the standard immunization schedule, either before or after transplantation [7]. A good immunization status is paramount in SOT recipients, since they are at higher risk of invasive HiB infection, in particular until the age of 5 years.

### 3.5. Hepatitis A

All SOT seronegative candidates should be considered for the vaccinations against Hepatitis A (HAV), but this is particularly relevant in candidates for liver transplant, due to the increased risk of severe Hepatitis A infection in patients affected by underlying liver diseases [16].

The HAV vaccine can be administered from the age of 12 months, with a two-dose schedule (see Table 1). 

In healthy children, the protective antibody response is reached in 97% of cases after the first dose and in 100% of cases after the second dose. In paediatric SOT recipients, the HAV vaccine is considered safe, but in this case, a high degree of heterogeneity and a strong decline in antibody titres have been observed after vaccination [1,7]. 

### 3.6. Hepatitis B

All SOT candidates, in particular liver and renal, should receive HBV vaccination before transplantation if they are not immune yet. In fact, these recipients may present a rapid and severe progression of this infection, precipitated by immunosuppression, in case of either reactivation of latent or newly acquired HBV infection.

Moreover, the pre-transplant response to HBV immunization has a high variability and depends on the underlying disease. 

For example, in liver transplant candidates, the immune response shows an inverse correlation with the severity of liver disease; in patients with advanced cirrhosis, the sero-conversion after HBV vaccination is low, with a range from 16 to 28%. 

In patients receiving HBV immunization after a liver transplant, it is demonstrated that the antibody response is also low, ranging from 7 to 23%; after repeated doses, it raises to 32% to 36% in SOT recipients, compared to 90% to 95% of healthy controls [21].

In another retrospective study, evaluating 56 patients receiving small bowel transplantation (associated or not to liver), seroprotection was best achieved in children receiving HBV vaccination before transplant and in the group receiving both small bowel and liver graft; moreover, the protective immunity tends to wane after 2 years post-transplant, leading to the need for booster doses [27].

Therefore, immunization can be performed either before or after transplantation and consists of three vaccine doses: the first two doses are administered at monthly intervals and a third dose is administered six months after the second (see Table 1). One month after the final dose, the measurement of anti-HBsAg antibody concentration can guide the following steps:

- Non-responder subjects (showing anti-HBsAg < 10 IU/L) require a new course of vaccination;
- Responder subjects need a re-assessment of antibody concentrations every 12 months. If HBsAg titres drop below 10 IU/L, a booster immunization dose should be administered [7].

In SOT candidates with an age ≥ 12 years, a combined HAV-HBV vaccine can be used [19]. 

Since vaccination trials for both HAV and HBV have been conducted mainly in liver transplant recipients, the results can only be extrapolated to other SOT types [1]. 

### 3.7. Meningococcal Vaccine

All SOT candidates and recipients should receive complete meningococcal immunization. 

The available meningococcal vaccines are able to target either serogroup B or the cluster of serogroups A, C, Y and W-135. Meningococcal vaccinations against MEN B have different schedules in different countries (see Table 1). Meningococcal vaccination against serogroups A, C, Y and W can also be administered with different vaccine formulations at different ages according to different schedules depending on different countries (see Table 1) [7,20].

### 3.8. Pneumococcal Vaccine

SOT candidates have a 12.8-times increased risk to develop invasive pneumococcal disease (IPD) in comparison to healthy subjects. For this reason, it is essential to guarantee to this population the best immunization against *Streptococcus pneumoniae*.

For the paediatric age, two vaccine types are available: a 23-valent pneumococcal polysaccharide vaccine (PPV23) and a 13-valent pneumococcal conjugate vaccine (PCV13). Recently, the new formulation of the 15-valent pneumococcal conjugate vaccine (PCV15) is under approval and proposed in place of PCV13 in healthy children; no data are available on its use in paediatric SOT recipients [28].

Protein-conjugated vaccines, such as PCV13 and PCV15, are able to induce a T-cell-dependent response, leading to the production of antibodies of higher avidity and to the formation of memory B cells; on the contrary, PPV23 elicits a T-cell-independent response [20,25]. 

In childhood vaccination schedules, which can be extended to SOT candidates and recipients, the suggested program is the following:

- Children less than 2 years of age should receive a 13-valent conjugated vaccine.
- Children from 2 to 5 years of age should undergo the following schedule: two doses of PCV13, second dose > 8 weeks after the first one; in addition, children who are transplant candidates should receive PPV23 at least 8 weeks after completing PCV13 schedule.

For transplant candidates older than 5 years, if they were previously vaccinated with PCV, PPV23 should be used; if they are pneumococcal vaccine naïve, they should receive a dose of PCV-13 first, followed by a dose of PPV-23 at least 8 weeks later [16,21,25].

If the vaccination schedule starts or resumes after SOT, PCV13 can be administered 2 to 6 months after transplant; if the patient is ≥2 years old, he can receive one dose of PPV23 2 to 6 months after SOT, with the timing based on the patient’s level of immunosuppression.

Overall response to anti-pneumococcal vaccination in SOT ranged from 32% to 100%, with comparable responses to the healthy population [19].

### 3.9. HPV Vaccine

Male and female SOT candidates, aged 11–26 years, can be immunized with a three-dose Human Papilloma Virus (HPV); if the vaccination schedule is not completely achieved before the transplant, it can be resumed with additional doses, starting 3–6 months post-SOT [21,26].

There are few available data on the immunogenicity of the HPV vaccine in the post-transplant setting. In a study performed on 23 kidney transplant patients (age 9–21 years) the antibody responses observed ranged from 33% to 80%, depending on HPV type; transplant recipients showed lower antibody titres than non-transplanted patients with chronic kidney disease. Differently, in another study on 20 adolescent liver and kidney recipients, the antibody responses were equivalent to healthy controls after the administration of the quadrivalent HPV vaccine [20,25].

### 3.10. Influenza

A seasonal inactivated influenza vaccination (IIV) should be given to patients listed for transplantation older than 6 months, as well as to their family members, close contacts, healthcare workers and also to family members of infants less than 6 months of age. 

In SOT recipients, annual vaccination with IIV is recommended, starting 3 to 6 months after transplantation; in case of a community influenza outbreak, IIV can be anticipated and administered ≥1 month after transplantation. 

As an example, a study on 187 paediatric kidney transplant recipients, 125 of whom received their influenza vaccine within the first year after transplant, demonstrated that this vaccine is safe, since no difference in graft survival or rejection was observed between the vaccinated and unvaccinated group [29]. 

Compared to healthy controls, in SOT patients there is overall a 10% to 16% lower response rate. 

The live attenuated influenza vaccine is contraindicated in all immunocompromised persons, including SOT recipients [1,16,19].

### 3.11. COVID-19

In children, SARS-CoV-2 infection usually causes a mild disease, with significantly lower morbidity and mortality as compared to the adult population. However, cases of severe disease, characterized by respiratory failure or shock during acute infection, are described in children as well. Moreover, the post-infectious, immune-mediated multi-system inflammatory syndrome (MIS-C) associated with SARS-CoV-2 highlights the fact that COVID-19 and its consequences can also be a concern in children [30,31,32]. The COVerAGE database (a global demographic database of COVID-19 cases and deaths) reported that, among the 4.4 million COVID-19 deaths, 0.4% have occurred in paediatric age [33].

The few available studies on COVID-19 disease among paediatric SOT recipients show that this group of patients, in comparison with their immunocompetent peers, does not show any significant difference in terms of disease severity, need for hospitalization or mortality [31,32,34].

On this basis, in SOT candidates, COVID-19 vaccination should ideally be completed at least 2–4 weeks before the transplantation, whenever possible, to prevent the risk of impaired immunogenicity after-transplant. If the patients cannot receive COVID-19 vaccinations before transplant, they should be reassessed post-transplant.

COVID-19 vaccination of eligible paediatric SOT candidates and recipients and their household contacts can reduce individual infections and the associated morbidity and can prevent COVID-19 spreading at a population level [33].

At present, childhood COVID-19 vaccination is characterized by a wide variation in the available formulations, approved ages and timing of approval around the world [33].

Extensive studies have been performed in adult SOT recipients to evaluate immunogenicity and safety of these new vaccines, while there are few data on the paediatric SOT population. 

In 2022, a comprehensive systematic review was published, including 21 papers, to evaluate how effective the COVID-19 current vaccines were in adult patients with underlying diseases causing immunosuppression, such as SOT recipients; the overall seropositivity in different transplant programs was less than 50% (44.9%) [35].

In another meta-analysis, including 20 studies on adult liver transplant patients from different countries, the observed humoral response rate after COVID-19 vaccination was 0.70, a result significantly lower than healthy controls, but higher in comparison to other organ transplants, such as kidney [36].

Other systematic reviews from adult SOT suggested a reduced ability to develop both humoral and cellular responses to COVID-19 vaccines, leading to the need for repeated booster doses to reach an adequate response rate [37,38].

Some small studies suggest that immunogenicity to mRNA-based COVID-19 vaccines in paediatric age may be comparable to that observed in adult SOT recipients. 

For instance, a prospective cohort study on 52 paediatric SOT (aged 12–18 years), without previous SARS-CoV-2 infection, has shown a 56.8% response after the first dose and 73.3% after the second dose. In another cohort of heart and lung transplant recipients (with mean age at transplantation of 14.6 +/− 7.24), a good safety profile of the vaccine BNT162B2 (Pfizer-BioNTech) was demonstrated; however, the antibody responses in SOT recipients were significantly lower than in healthy controls [39].

In a small study on 20 kidney transplanted patients, the vaccine response was 50% after the second dose and 75% after the third, with prednisone and MMF used as factors associated with poor immunologic response [40].

Adverse events (AEs) in vaccinated individuals were mostly mild to moderate: local AEs include injection site pain, redness and swelling; systemic AEs consist of fatigue, headache, sleepiness, myalgia, arthralgia, fever and nausea/vomiting.

Severe adverse events may include febrile seizures in children; anaphylaxis, Guillain–Barré syndrome, myocarditis/pericarditis and thrombosis with thrombocytopenia syndrome were rarely reported and mainly in adults [41,42].

Since it is expected that transplanted children will have a reduced antibody response to COVID-19 vaccination, it might be necessary to provide booster doses over time (see Table 1) [30].

## 4. Live Vaccinations: Pre- and Post-Transplant Indications

### 4.1. Measles, Mumps, Rubella and Varicella

In SOT candidates and recipients, primary chicken pox and measles infection can result in severe consequences, such as dissemination, respiratory failure and allograft rejection; for instance, untreated varicella in immunocompromised persons could be the cause of death in up to 7% of patients [43,44].

MMR and VZ seronegativity was observed in SOT candidates with a range between 7% to as high as 50%, depending on age, prior vaccination status and pre-transplant level of immunosuppression [20,25,45].

In a study of children listed for liver transplant, the pre-transplant seropositivity rates were 47% for measles, 49% for mumps and 68% for chickenpox [46,47].

MMR and VZ serology should be checked at the time of listing, and SOT candidates should be immunized as much as possible, balancing the benefit of live viral vaccines against the risk of delay in active listing. 

The standard vaccination schedule provides that live attenuated MMR and varicella vaccine (VV) are routinely given to healthy children, starting at ≥12 months of age [43,45].

This is because in very young infants, the presence of maternal antibodies interferes with the response to live vaccines; therefore, they are most effective after 1 year of age, when this passive immunization has waned [20,25].

MMR and VV, separated or combined, should be administered ≥4 weeks prior to transplant; ideally, two doses should be administered ≥3 months apart [45].

If needed, SOT candidates who are aged 6–11 months can receive these vaccinations on an accelerated schedule; the second dose can be provided 4 weeks after the first; if transplantation is delayed, the vaccine should be repeated at 12 months [13].

In the practical setting, pre-transplant live vaccinations are frequently not performed because patients are either too young or considered too ill, or because of insufficient time before the planned SOT; for instance, less than 30% of liver transplant recipients 6–12 months of age have received live vaccines [46]. Furthermore, in children vaccinated before SOT, antibodies may wane over time, with significant loss of immunity.

To improve the protection of SOT candidates and recipients, vaccinations of household members for MMR and VV is also recommended and considered safe. A theoretical risk of transmission exists, in case the vaccinated person develops skin lesions, but so far there is no evidence of transmission of vaccine-derived measles, mumps and rubella infections [43].

Due to safety concerns, to date most of the transplanted patients did not receive live-attenuated vaccines; for this reason, they remain susceptible individuals and may need post-exposure prophylaxis, such as specific immunoglobulins or antivirals; in this case, the efficacy in disease prevention is variable [43,45].

Considering MMR and VV administration after SOT, studies have been performed almost exclusively in paediatric liver and kidney transplant recipients [48].

In these studies, different timing, different patterns of sero-conversions and different needs for booster doses were evaluated.

In a study on 39 paediatric recipients receiving living-donor liver transplantation, post-transplant immunizations were performed for measles, rubella, mumps and varicella; the observed seroprotection rates were 44%, 70%, 48% and 32%, respectively. The recipients with primary vaccine failure after first vaccination received a second dose, leading to seroprotection rates for measles, rubella, mumps and varicella of 100%, 50%, 71% and 50%, respectively [43].

In another study, 90 paediatric patients (age < 18 yo) were enrolled; patients unprotected for measles and fulfilling specific safety criteria (low level of immunosuppression and a sufficient lymphocyte count) were eligible for MMR immunization. 

At inclusion, 51% percent of children were not seroprotected against measles, although 39% of them had previously been vaccinated. Additionally, 93% of patients reached seroprotection 4 weeks after the first dose; the seroresponse to a two-dose schedule thus reached 98%. 

A third study, published in 2015, considered 196 immunizations, administered to 48 paediatric post-LDLT recipients: the sero-conversion rates after the first dose of measles, rubella, varicella and mumps were 100%, 100%, 70% and 75%, respectively [49].

Lastly, a study on 77 liver-transplanted children showed that 39 were seronegative for VZV; 36 were suitable for immunization and vaccinated against VZV. After immunization, none of the patients reported clinical or biological episodes of graft rejection; half of them had local reactions and two-thirds had some systemic reactions, similar to what is described in the general healthy population. To obtain a long-lasting sero-conversion, most of the VZV naïve patients needed two doses of the vaccine; this proportion of non-seroresponders after two doses is higher than in healthy children. Therefore, 21.9% of children required a third dose to develop a good antibody response to vaccination; after a maximum of three doses, all children were able to reach protective antibody levels [44].

The general conclusion arising from these different studies was that live vaccines can be safe in a selected population of SOT recipients, and are well tolerated, with minor adverse events, similar to those observed in the healthy paediatric population; moreover, SOT recipients can reach a good seroprotection, but with the need of repeated immunizations to ensure sustained response. Finally, no cases of infection due to the attenuated vaccine strain were reported [17,18,44,47,48,49,50].

According to these results, a consensus was held in 2018, leading to specific indications for the administration of MMR and VV after SOT under certain circumstances, defined by the following criteria:

- Renal and liver transplant, more than one year post-transplant, 2–6 months post-episode of acute rejection, AND “minimum immune suppression” AND “minimum immune criteria”.
- The “minimum immune criteria” are defined as follows: absolute lymphocyte count >1500 (children ≤ 6 yo), or >1000 (children > 6 yo) cells/μL, CD4 > 700 (children ≤ 6 yo) or >500 (children > 6 yo), cells/μL; normal IgG levels; consider the ability to produce protective antibodies to inactivated vaccines prior to administration of live viral vaccines.
- The “minimum immune suppression” is defined as follows: steroid < 2 mg/kg/day or total cumulative < 20 mg/day, tacrolimus < 8 ng/mL or cyclosporine < 100 ng/mL.

Some other criteria were defined to suggest caution in a group of patients for whom the evidence of safety and efficacy of live vaccination is unclear; in particular:

- Patients who are receiving mycophenolate mofetil (MMF);
- Patients who have received T-cell depleting agents (for ATG wait 12 months, for Alemtuzumab wait 24 months);
- Patients who have received Rituximab (wait at least 12 months);
- Patients with persistently elevated Epstein Barr Virus (EBV) viral load;
- Patients who underwent total timectomy;
- Patients who achieved a condition of clinical operational tolerance, with all immunosuppressive treatments withdrawn; it is unclear the safety of live vaccination in triggering a rejection episode [18,24].

More recently, following the criteria established by the previous consensus, a cohort of 31 children after heart transplantation were vaccinated against VZV, with a good early sero-conversion (94% of patients after 6 months) and a mild adverse event profile (32% of patients experimenting rash or local site reaction) [51].

Despite all these results, post-transplant live vaccines are still not offered by all centres, mainly because of concerns about safety and lack of efficacy data. 

Lastly, no data are available on combined MMR-V vaccines, including all viral strains in the same preparation; for this reason, it is not recommended in SOT recipients [17,24].

### 4.2. Rotavirus

Although this vaccine has not been studied in transplant recipients, it could in theory be administered to SOT infant candidates, according to the age criteria, taking into account that viral antigens persist in stools at least up to 28 days post-vaccination [16].

## 5. Discussion and Conclusions

Even in the era of continuous progress in the medical management of transplanted patients, still infectious diseases are an important cause of morbidity and mortality in paediatric SOT recipients compared to immunocompetent subjects. 

Paediatric patients with organ failure are at higher risk of infections, both before and after transplantation, due to the age-related immaturity of their immune system and to the effect of immunosuppressive therapy. 

One of the most important challenges in paediatric SOT is the balance between the risk of rejection/graft dysfunction on one side and the morbidity and mortality secondary to infectious diseases on the other side. 

Vaccination is the active intervention with the best results, in terms of efficiency and cost–benefit, to prevent infectious diseases. 

Since the need for transplantation at paediatric age is frequently in the first years of life, it is likely that children may lack previous immunity from natural exposure and may not have had time to finish their primary immunization schedule by the time of transplant.

For this reason, the “ideal” strategy is to timely verify the vaccination status of all the patients with advanced diseases, aiming at promptly identifying and filling any possible immunity gaps before SOT. The maximum effort should be made to implement the vaccination program in these patients, even by accelerated schedules, to obtain the best immunization before the transplant. 

Moreover, it is necessary to continue or complete the vaccination programs after the transplant, since these patients are always more exposed to all VPIs and other infections. 

Finally, to optimize the protection from infections for paediatric SOT recipients, vaccination strategies are also essentials in close contacts, such as family members and healthcare professionals. This population should receive all the recommended immunizations, in particular MMR, Varicella and SARS-CoV-2, as well as annual inactivated influenza vaccine, in order to minimize the exposure of SOT recipients to wild-type viruses. 

This review has several limitations. Firstly, the majority of studies conducted at paediatric age are limited in their sample size. The robust literature on vaccinations in SOT candidates and recipients comes from adult patients and, as usual, these results can only partially be shared in the paediatric setting, with its special characteristics; smaller studies involving only children have limited statistic power and the results cannot be easily generalized. Moreover, most of the paediatric studies were performed in liver and kidney transplant, and only few reports involve other organs (heart, lung and bowel). This is also a limitation, since the findings from some organs may not apply to all, and any extrapolation needs to be cautious.

A special consideration shall be given to the recommendation on COVID-19 vaccines, since the knowledge is still in progress, and the recommendations might be updated over time, as more data will be available. Furthermore, the present studies necessarily cover a short time period to assess both safety and antibody responses, in healthy population as well as in SOT candidates and recipients; further studies will be mandatory to assess SARS-CoV-2 long-term protection and outcomes in paediatric patients.

Finally, the majority of the studies were performed in North America and Europe, thus considering a limited community, representing approximately 17% of the world’s population. It might be challenging to define standardized protocols that can be applied to the entire global population. The WHO vaccination recommendations partially address this problem by considering the needs of various countries based on their epidemiology and resources. However, the development of standardized guidelines for paediatric SOT candidates and recipients that can be valid worldwide remains a challenge.

## Figures and Tables

**Table 1:** Paediatric SOT candidate and recipient immunization recommendations: indications and schedules.

Type of Vaccine	Minimum Age at First Dose	Standard Schedule	Accelerated Schedule	Recommended after Transplant
Inactivated Vaccines				
Diphtheria/Tetanus/Pertussis	6 weeks	*WHO schedule*: - The primary series includes 3 doses (with interval between doses: 4–8 weeks) 3 booster doses are administered as follows: - At 12–23 months DTP-containing vaccine; - At 4–7 years only Td-containing vaccine; - At 9–15 yrs only Td-containing vaccine. *CDC schedule:* 5-dose series are administered at ages 2, 4, 6, 15–18 months, 4–6 years.	The minimum interval between 1st and 2nd dose: 4 weeks; between 2nd and 3rd dose: 4 weeks; between 3rd and 4th dose: 6 months and after age of 12 months; between 4th and 5th dose: 6 months and after age of 4 yo.	Yes
Inactivated Polio (IPV, applied to countries in polio-free regions with a very low risk of importation and sustained high routine immunization coverage)	6 weeks	*WHO schedule:* The primary series includes 3 doses (with interval between doses: 4–8 weeks); an IPV booster (6 months after 3rd dose) is needed when 1st dose is given at <8 weeks. *CDC schedule:* 4-dose series are administered at age 2 months, 4 months, 6 through 18 months, 4 through 6 years.	Minimum interval between 1st and 2nd dose: 4 weeks; between 2nd and 3rd dose: 4 weeks and after age of 6 months; between 3rd and 4th dose: 6 months and after age of 4 yo.	Yes
Haemophilus Influenzae B (HiB)	6 weeks	*WHO schedule includes 3 options:* 3 primary doses without a booster *OR * 2 primary doses plus a booster *OR * 3 primary doses with a booster Interval between doses: 4 weeks in case where 3 primary doses are given; 8 weeks in case where 2 primary doses are given. Booster doses: at least six months after completion of the primary series. Single dose if >12 months of age. Not recommended for children > 5 yrs. *CDC schedule:* 4-dose series, including 3-dose primary series at age 2, 4 and 6 months, followed by a booster dose at age 12–15 months *OR* 3-dose series, including 2-dose primary series at age 2 and 4 months, followed by a booster dose at age 12–15 months.	Minimum interval between 1st and 2nd dose: 4 weeks; between 2nd and 3rd dose: 4 weeks; between 3rd and 4th dose: 8 weeks and after age of 12 months.	Yes
HBV	At birth	*WHO schedule includes 2 options:* 3-doses schedule, with first dose given at birth and second and third doses given at the same time as the first and third doses of DTP-containing vaccine; *OR* 4-doses schedule, where a monovalent birth dose is followed by 3 doses, usually given with other routine infant vaccines. The interval between doses should be at least 4 weeks. *CDC schedule:* 3-dose series at age 0, 1–2, 6–18 months. Infants who did not receive a birth dose should begin the series as soon as possible; in this case: dose interval 1st–2nd dose: 4 weeks; dose interval 2nd–3rd dose: 8 weeks and at least 16 weeks after first dose. Minimum age for the final dose: 24 weeks.	Minimum interval between 1st and 2nd dose: 4 weeks; between 2nd and 3rd dose: 8 weeks and after 24 weeks of age.	Yes
HAV	≥12 months	*WHO schedule:* 1 or 2 doses; interval between 1st and 2nd dose: 6–18 months (max around 4–5 years) *CDC schedule: * 2-dose series at age 12–23 months (minimum interval: 6 months)	Minimum interval between 1st and 2nd dose: 4 weeks.	Yes
HPV	9 years	*WHO schedule:* 1–2 doses in girls at 9–14 yo; Interval between doses: 6–12 months. *CDC schedule: * 2- or 3-dose series, depending on the age at first vaccination: Age 9–14 years: 2-dose series at 0, 6–12 months (minimum interval: 5 months). Age 15 years or older: 3-dose series at 0, 1–2 months, 6 months.	Minimum interval between 1st and 2nd dose: 4 weeks; between 2nd and 3rd dose: 12 weeks.	Yes
MENACVY	2-9-23 months (according to different vaccine formulations)	*WHO schedule:* One single dose to individuals ≥2 years. It is also approved for children 9–23 months of age and given as a 2-dose series, 3 months apart, beginning at the age of 9 months. *CDC schedule:* 2-dose series at age 11–12 years and 16 years. Only in particular conditions (functional asplenia, HIV infection, persistent complement component deficiency), different schedules, starting from 2 months of age.	First dose at 9 months; interval between 1st and 2nd dose: 12 weeks.	Yes
MENB	3 months	*WHO schedule: * This vaccine is not included. *CDC schedule: * Adolescents 16–18 yo: 2-dose series, at least 1–6 months (according to different vaccine formulations) *In some European Countries: * 2-dose series at 3–5 months of age, followed by a booster dose at 15 months of age. Minimum interval between 1st and 2nd dose: 2 months. Minimum interval between 2nd dose and booster: 6 months.	/	Yes
Pneumococcal disease	6 weeks	*WHO schedule: * **PCV 13** vaccine, including 2 options: - 2 primary doses at an interval of ≥8 week, followed by a booster dose at 9–18 months of age. - 3 doses, each 4 weeks apart. *CDC schedule: * 4-dose series at 2, 4, 6, 12–15 months (**PCV13** or **PCV 15**) **For PPV23 indications: see text**.	Minimum interval between 1st and 2nd dose: 4 weeks; between 2nd and 3rd dose: 4 weeks; between 3rd and 4th: 8 weeks. If between 12 and 24 months and unvaccinated: give 3 doses, 8 weeks apart; If between 24 months and 5 yo and unvaccinated, give 1 dose.	Yes (see text for details)
SARS-CoV-2	6 months	*WHO schedule*: Not included (different approach in different countries, according to vaccine availability) *CDC schedule: * Age 6 months–4/5 years: 2-dose series at 0, 4–8 weeks (bivalent Moderna, 0.25 mL/0.25 ug) *OR* 3-dose series at 0, 3–8 weeks, 8 weeks (2 months) after dose 2 (bivalent Pfizer-BioNTech, 0.2 mL/0.3 ug); Age > 5 years: 1 dose bivalent Moderna (6–11 yo dose 0.25 mL/25 ug; ≥12 yo dose 0.5 mL/50 ug) *OR * 1 dose bivalent Pfizer-BioNTech (age 5–11 yo dose 0.2 mL/10 ug; age ≥ 12 yo dose 0.3 mL/30 ug) In adolescents ≥12: Novavax 2-dose primary series (separated by at least 3–8 weeks)	/	*CDC schedule* for immunocompromised persons (such as transplant recipients): Age 6 months–4 years: 3-dose series at 0, 4, 8 weeks (Moderna) *OR* 3-dose series at 0, 3, 11 weeks (Pfizer-BioNTech); Age 5–11 years: 3-dose series at 0, 4, 8 weeks (Moderna) *OR* 3-dose series at 0, 3, 7 weeks (Pfizer-BioNTech) Age 12–18 years: 3-dose series at 0, 4, 8 weeks (Moderna) *OR* 2-dose series at 0, 3 weeks (Novavax) *OR* 3-dose series at 0, 3, 7 weeks (Pfizer-BioNTech) *Booster dose*: 1 more dose at least 2 months following the last recommended dose.
Influenza inactivated vaccine (inactivated tri- and quadrivalent)	6 months	*WHO and CDC schedule:* 6 mo–8 yo: 2 doses, 4 weeks interval between doses. ≥9 years: 1 dose. Revaccinate annually: 1 dose only.	Interval between 1st and 2nd dose: 4 weeks.	Yes
LIVE vaccines				
MMR (Measles/Mumps/Rubella)	9–12 months (WHO) 12 months (CDC)	*WHO schedule:* 2 doses, interval between doses: 4 weeks (min) *CDC schedule: * 2-dose series at age 12–15 months, 4–6 years	Anticipate 1st dose at 6 months; interval between 1st and 2nd dose: 4 weeks.	Only in selected cases (see text)
VZV	12–18 months	*WHO schedule:* 1–2 doses; interval between 1st and 2nd dose from 4 weeks to 3 months, according to manufacturer recommendations *CDC schedule:* 2-dose series at age 12–15 months, 4–6 years. Dose 2 may be administered as early as 3 months after dose 1.	Anticipate 1st dose at 6 months; interval between 1st and 2nd dose: 4 weeks.	Only in selected cases (see text)
Rotavirus	6 weeks	*WHO schedule:* 2–3-dose series (depending on product); interval between doses: 4 weeks (min) Vaccination of children >24 months of age is not recommended. *CDC schedule:* 2-dose series at age 2 and 4 months *OR* 3-dose series at age 2, 4 and 6 months (depending on product). maximum age for the final dose: 8 months	Minimum age: 6 weeks. Interval between 1st and 2nd dose: 4 weeks. Interval between 2nd and 3rd dose: 4 weeks.	No

Data from WHO schedule: Summary of WHO Position Papers—Recommended Routine Immunizations for Children. Update March 2023. CDC schedule recommendation—USA 2023. Abuali et al. [6] Paediatr Transplant 2011—Table 1.
